# Loss of quiescence and self-renewal capacity of hematopoietic stem cell in an in vitro leukemic niche

**DOI:** 10.1186/s40164-016-0062-1

**Published:** 2017-01-10

**Authors:** Natalia-Del Pilar Vanegas, Jean-Paul Vernot

**Affiliations:** Cellular and Molecular Physiology, Biomedical Research Institute, Faculty of Medicine, Universidad Nacional de Colombia, Bogotá, D.C. 111321 Colombia

**Keywords:** Leukemic niche, Hematopoietic stem cells, Mesenchymal stem cells, Quiescence, Self-renewal, Proliferation, Microenvironment

## Abstract

**Background:**

Leukemic and mesenchymal stem cells interact in the leukemic microenvironment and affect each other differently. This interplay has also important implications for the hematopoietic stem cell (HSC) biology and function. This study evaluated human HSC self-renewal potential and quiescence in an in vitro leukemic niche without leukemic cells.

**Methods:**

A leukemic niche was established by co-culturing mesenchymal stem cells with a fresh conditioned medium obtained from a leukemic (REH) cell line. After 3 days, the REH-conditioned medium was removed and freshly isolated CD34+ at a density of up to 100,000 cells/ml were added to the leukemic niche. CD34+ cell evaluations (cell cycle, self-renewal gene expression and migration capacity) were performed after 3 further days of co-culture. Additionally, we preliminary investigated the soluble factors present in the leukemic niche and their effect on the mesenchymal stem cells. Statistical significance was assessed by Student’s t test or the nonparametric test Kolmogorov–Smirnov.

**Results:**

By co-culturing normal mesenchymal stem cells with the REH-conditioned medium we showed that hematopoietic stem cells, normally in a quiescent state, enter cell cycle and proliferate. This loss of quiescence was accompanied by an increased expression of Ki-67 and c-Myc, two well-known cell proliferation-associated markers. Two central regulators of quiescence GATA2 and p53 were also down regulated. Importantly, two genes involved in HSC self-renewal, Klf4 and the histone–lysine *N*-methyltransferase enzyme Ezh2, were severely affected. On the contrary, c-Kit expression, the stem cell factor receptor, was upregulated in hematopoietic stem cells when compared to the normal niche. Interestingly, mesenchymal stem cells incubated with the REH-conditioned medium stopped growing, showed a flattened morphology with the appearance of small vacuoles, and importantly, became positive for the senescence-associated beta-galactosidase activity. Evaluation of the leukemic-conditioned medium showed increased IL-6 and IL-8, suggesting that these cytokines could be responsible for the observed changes.

**Conclusions:**

Our results showed that quiescence and self-renewal are severely affected in this leukemic niche. This in vitro leukemic niche, established without leukemic cells, will facilitate HSC gene expression evaluation and the development of therapeutic agents aimed to neutralize soluble factors and the cell signaling pathways involved in HSC alterations.

**Electronic supplementary material:**

The online version of this article (doi:10.1186/s40164-016-0062-1) contains supplementary material, which is available to authorized users.

## Background

Hematopoietic stem cells (HSC) are characterized by their self-renewal and multipotent differentiation capacities. These properties are necessary for the maintenance of the HSC pool and important in the development and regeneration of the hematopoietic system under normal and stressful conditions [1–3]. HSC reside in specialized niches [4] in the bone marrow (BM) with which they interact continuously, influencing these properties [5, 6]. Multiple cellular types, soluble factors and extracellular matrix components form this niche [7, 8]. Nevertheless, a predominant role is played by the mesenchymal stem cells (MSC) due to their multilineage differentiation capacity and their regulation of HSC function [9, 10].

Under homeostatic conditions, most HSC exist in the G0 phase of the cell cycle with signals from the niche used to preserve a quiescent state as a protective mechanism against cell damage and exhaustion [6]. Other signals stimulate the appearance of cell progenitors and thus cell cycle entry and proliferation [11]. In purified mouse stem and progenitors cells the molecular network regulating self-renewal, differentiation and the maintenance of quiescence has been identified [12]. These authors have shown that the long-term engraftment potential resides mainly in the G0 cell fraction. Therefore, a quiescent state is fundamental for preserving key functional features necessary for transplantation. In humans, it has been shown that HSC in the G0 phase have better adherence to MSC [13] and we have previously shown that primitive HSC in co-culture with MSC induce a VCAM-dependent pro-migratory phenotype [14], suggesting that the G0 cell fraction has better BM homing efficiency. Importantly, the relationship between cell cycle regulators (cytokines, cyclin and cyclin-regulators, transcription factors, microRNA, etc.) and HSC self-renewal has been partially elucidated [5, 15].

The extracellular signals responsible for HSC maintenance and survival are well known. SCF/c-Kit, Ang-1/Tie1/2 and TPO/c-MPL signaling is essential for maintaining HSC quiescence, survival and function [1, 16–18]. Also, it has been reported that the SDF-1/CXCL12 chemokine acts not only as an essential chemoattractant for HSC homing but also as a regulator of HSC quiescence [19]. Likewise, SDF-1/CXCL12 is an inhibitor of human long-term culture-initiating cell cycling and therefore its reduction in the BM induces cell cycling [20]. Abnormal stimulation of these receptors could induce HSC cycling and exhaustion [21]. HSC proliferation can also be induced in situations of hematopoietic challenge, i.e., infection/inflammation, senescence, leukemia, chemotherapy, irradiation and BM transplantation, among others [22–24].

Likewise, early work has shown that BM stimuli are also essential for leukemic cell growth and expansion [25] and that leukemic cell growth affects HSC function both in vivo and in vitro [26]. Recently, it has also been shown in mouse models, that the BM niche protects leukemic cells against chemotherapy, and that the leukemic-induced decrease in SDF-1 levels is responsible for impaired human CD34+ homing and retention [27, 28]. All these results suggest that the microenvironment that supports leukemic cells and affects HSC properties [12] could be a valid target for chemotherapeutic intervention.

In the present study, we were interested in the evaluation of human HSC self-renewal potential and cell cycle regulation in an in vitro leukemic niche. By incubation of MSC with a leukemic-conditioned medium, obtained from an acute lymphocytic leukemia pre-B (ALL-B) cell line (REH), we were able to study gene expression of some important molecules involved in HSC function. In this system, HSC showed increased cell proliferation and gene expression changes compatible with loss of self-renewing potential. These alterations are equivalent to the observed reduced hematopoiesis, accompanied by an increase in progenitors and differentiated cells, seen in patients with ALL [29].

## Methods

### Isolation of CD34+ from umbilical cord blood (UCB)

UCB samples were obtained from normal full-term deliveries. Mononuclear cells (MNC) were isolated by Ficoll-Hypaque density gradient centrifugation and CD34+ cells were purified using a MACS CD34+ isolation kit (Miltenyi Biotec, Auburn, CA, USA). Cell purity was evaluated by flow cytometry using allophycocyanin anti-CD34 (Clone AC136, Miltenyi Biotec) and cell viability by Trypan blue dye exclusion.

### Isolation and characterization of BM-MSC

BM-MSC were isolated from BM aspirates of healthy young donors. MSC were cultured, expanded and characterized according to the criteria established by the *International Society for Cell Therapy* (cell morphology, expression of cell surface markers and the ability to differentiate into osteoblasts, chondrocytes and adipocytes) [30]. After the 3rd passage, adherent cells were trypsinized and labeled with the following monoclonal antibodies: PE mouse anti-human CD73 (clone AD2, BD Pharmingen), APC mouse anti-human CD105 (clone 43A4E1, Miltenyi Biotec), PerCP mouse anti-human CD45 (clone 2D1, BD Biosciences), FITC mouse anti-human CD90 (clone F15-42-1, Abcam), APC mouse anti-human CD34 (clone AC136, Miltenyi Biotec) and FITC mouse anti-human CD44 (clone MEM-85, Invitrogen). Data were acquired using a FACSAria II flow cytometer (BD Biosciences, San Jose, CA, USA). FACS Diva software, CellQUEST PRO software, FlowJo, and Paint-a-Gate software (BD Biosciences) were used for data analysis.

The mesenchymal lineages differentiation capacity of MSC was determined using specific staining and microscopic observation, as previously described [31]. Briefly, 3rd passage 2 × 10^4^ MSC were cultured in a 24-well plate in IMDM until they reached confluence. For adipogenic differentiation, cells were cultured for 3 days in induction medium (MEMα supplemented with 10% FBS, 1 mM dexamethasone, 0.5 mM isobutylmethylxanthine, 200 μM indomethacin, and 10 μg/ml insulin, all reagents from Sigma Aldrich) followed by incubation in maintenance medium (MEMα, supplemented with 10% FBS and 10 μg/ml insulin) for 3 days, and these treatments were repeated twice. Osteogenic differentiation was induced by incubation with the induction medium MEMα supplemented with 10% FBS, 100 nM dexamethasone, 0.2 mM ascorbic acid 2-phosphate, and 10 mM β-glycerophosphate (all reagents from Sigma Aldrich) for 2 weeks. Finally, for chondrogenic differentiation, cells were plated and cultured in a chondrogenic induction medium (MEMα and 10 ng/ml TGFβ-1, Sigma Aldrich) for 2 weeks. Cells were washed with PBS 1X, formalin fixed, and stained with 0.35% Oil Red O solution (Sigma Aldrich) for adipogenic differentiation, alkaline phosphatase (AP staining kit, Chemicon Int.) for osteogenic differentiation, or with 0.1% Safranin O (Sigma Aldrich) for chondrogenic differentiation. Cells were examined under an inverted microscope (Nikon, Model TS-100) and photographed with a Canon Power Shot A460, Zoom Browser EX software. Characterized MSC were expanded, frozen and used for the different experiments in passages 3–5.

### REH-conditioned medium (REH-CM) preparation

2.5 × 10^5^ REH cells/ml were cultured in RPMI Glutamax-I (GIBCO, Invitrogen) supplemented with 1% sodium pyruvate, 1% MEM non-essential amino acid solution 100× and 1% FBS for 24 h at 37 °C and 5% CO2 in 75 cm^2^ culture flask. Next, REH cells were centrifuged at 500*g* × 7 min, the supernatant was collected and filtered through a 0.22 µm pore membrane filter and used fresh in all experiments.

### Establishment of the leukemic niche (LN*)*

BM-MSC at 80% confluence were co-cultured for 3 days with fresh REH-CM (with a re-feeding after 1.5 days). After REH-CM addition MSC stopped dividing and did not further increase cell number (not shown). After 3 days, the REH-CM was gently removed from the wells and freshly isolated CD34+ at a density of up to 100,000 cells/ml were added to these MSC. For the establishment of the so-called normal niche (NN), MSC were set at a lower density in order to have 80% confluence after 3 days of culture [31]. The idea was to have both NNs and LN with MSC at 80% confluence when the CD34+ cells were added. Freshly isolated CD34+, from the same UCB used for the LN, were layered over MSC at the same 80% confluence but at two fetal bovine serum (FBS) concentration, 10% (NN10) or 1% (NN1). A fraction of these CD34+ cells was kept for evaluation in each experiment (freshly isolated CD34+ cells). CD34+ cell evaluation in the different niches was done after 3 days of the co-culture.

### Cytometric bead array (CBA) determination of cytokines

For the determination of the human pro-inflammatory cytokines concentration in the supernatant of the different cultures we have used the cytometric bead array kit (Becton–Dickinson Biosciences) for human inflammatory cytokines, following the manufacturer’s instructions. Supernatants were obtained from MSC after 3 days of culture in IMDM supplemented with 10% FBS, 1% sodium pyruvate, 1% MEM Non- Essential Amino Acids (GIBCO, Invitrogen); MSC cultured in the same conditions except for 1% FBS; REH cells cultured in RPMI 1640 supplemented with 10% FBS, 1% sodium pyruvate, 1% MEM non-essential amino acids (GIBCO, Invitrogen); REH-CM prepared as indicated above; and co-cultures of MSC with REH cells, and MSC cells with REH-CM. Cytokines concentration was evaluated by flow cytometry (FACSAriaTM II). Flow Jo and FCAP Array v3.0 Software (Becton–Dickinson Biosciences) were used for data analysis.

### Cell cycle analysis by flow cytometry

For cell cycle analysis, freshly isolated CD34+ cells and CD34+ cells obtained from both the NN and the LN after co-culturing with MSC for 3 days were fixed with 4% formaldehyde and permeabilized with 0.1% Triton X-100. The cells were stained for 30 min at room temperature with the monoclonal antibody APC-conjugated anti-human Ki67 (clone Ki67, Biolegend) and/or incubated with Hoechst 33342 (2′-[4-ethoxyphenyl]-5-[4-methyl-1-piperazinyl]-2,5′-bi-1*H*-benzimidazole trihydrochloride trihydrate) (from Invitrogen) (2n DNA content, G0/G1; >2n DNA content, S-G2-M) for 45 min at 37 °C. Finally, the supernatants were removed and cells were resuspended in PBS 1X for flow cytometry evaluation (FACSAriaTM II, BD Biosciences). FlowJo and FCS Express Flow Cytometry Data Analysis Softwares v5.0 (BD Biosciences) were used for data analysis.

### Gene expression analysis by real time-PCR-SYBR green

Freshly isolated CD34+ cells and CD34+ cells obtained from the NN or the LN were washed in PBS and total RNA was extracted with TRIzol reagent (Invitrogen). The RNA obtained was quantified by fluorogenic methods (NanoDrop 2000C, Thermo Scientific) and stored at −70 °C until analysis; A qualitative assessment by 1% agarose gel electrophoresis was performed. Eventual contamination by genomic DNA was removed by using the DNAse I Kit (Invitrogen). Reverse transcription was performed using the High Capacity Kit (Applied Biosystems) from 1000 ng RNA. qRT-PCR was performed on a 7500 real time PCR Systems (Applied Biosystems^®^) with the SYBR Green Master Mix (Applied Biosystems). The reactions were done in triplicate for each sample and in three independent experiments. The total reaction volume was 20 μl (including 0.5 μl cDNA, SYBR Green, and 0.1 mM of primers, see Additional file 1: Table S1). Relative gene expression levels were normalized to RPS18 transcript levels and calculated using the 2^−∆∆CT^ method. A “screening” of 19 selected genes was performed. The sequences of the primers were determined using Basic Local Alignment Search Tool (BLAST) based on previous publications; Forward and reverse primers used are shown in Additional file 1: Table S1.

### Senescence-associated β-galactosidase (SA-βGAL) evaluation in MSC

Senescence-associated β-galactosidase (SA-βGAL) activity was evaluated in MSC after incubation with the REH-CM in the same conditions as described above. For this purpose we have used the cellular senescence assay kit (KAA002, Millipore). Briefly, MSC were seeded in duplicates in 24-well plates, washed twice with PBS 1X, and then fixed with formalin for 10 min at room temperature. Next, MSC were washed with PBS 1X and incubated over-night at 37 °C with the β-gal substrate in an acidic buffer (pH 6.0) and examined with an inverted microscope (Nikon, Model TS-100) and photographed (Canon Power Shot A460, Zoom Browser EX software). The appearance of development of a perinuclear blue color was an indication of senescence.

### HSC migration evaluation

Transwell filters (Corning Costar) with 5 μm pore size, 6.5 mm diameter, inserted in a 24-well plate (Corning) were incubated for 1 h at 37 °C with the migration buffer (RPMI-1640 and 2% BSA). Then 5 × 10^4^ HSC from the NN10, NN1 and LN in 100 μl were added to the upper chamber. The lower chambers were filled with 600 μl of migration buffer containing recombinant human stromal derived factor-1 (rhSDF-1a, Miltenyi Biotec) at 100 ng/ml. After 4 h at 37 °C in a 5% CO_2_ incubator, cells that migrated to the lower chamber were harvested and counted with a FACSAria II flow cytometer (BD Biosciences, San Jose, CA, USA). The numbers of migrated cells were normalized to unstimulated controls (FACS acquisition was performed in duplicate in some experiments).

### Statistical analysis

All data are presented as mean + SEM. Statistical significance was assessed by Student’s *t* test or the nonparametric test Kolmogorov–Smirnov to compare cumulative distributions. *p* values <0.05 were defined as statistically significant.

## Results

### LN establishment with REH-CM

We have established an in vitro LN by incubation of MSC (Additional file 2: Figure S1A, B) with a REH-CM during 3 days. We have previously determined that this LN correctly simulated a LN set with REH cells in the same conditions (not shown). In this latter niche, REH cells were very difficult to be detached from the MSC and therefore accurately molecular evaluation of HSC after co-incubation was difficult specially if RNA extracts for gene expression analysis have to be prepared. Therefore the setting of a leukemic niche without leukemic cells was essential to study gene expression in HSC in a LN. Re-feeding with fresh REH-CM was done during the incubation period to ensure a proper and permanent exposure to soluble factors, simulating permanent REH cell secretion. After 3 days of incubation, the REH-CM was removed and freshly isolated CD34+ HSC (>95% purity and >95% cell viability) (Additional file 2: Figure S1C) were added for additional 3 days, after which HSC evaluations were performed. As controls, HSC co-cultured with MSC at the same cell confluence and in normal culture medium with 10% FBS (NN10) or 1% FBS (NN1) were performed. For comparison, HSC evaluations from freshly isolated HSC were also performed in each experiment.

### CD34+ cell proliferation in the LN

**Fig. 1:**
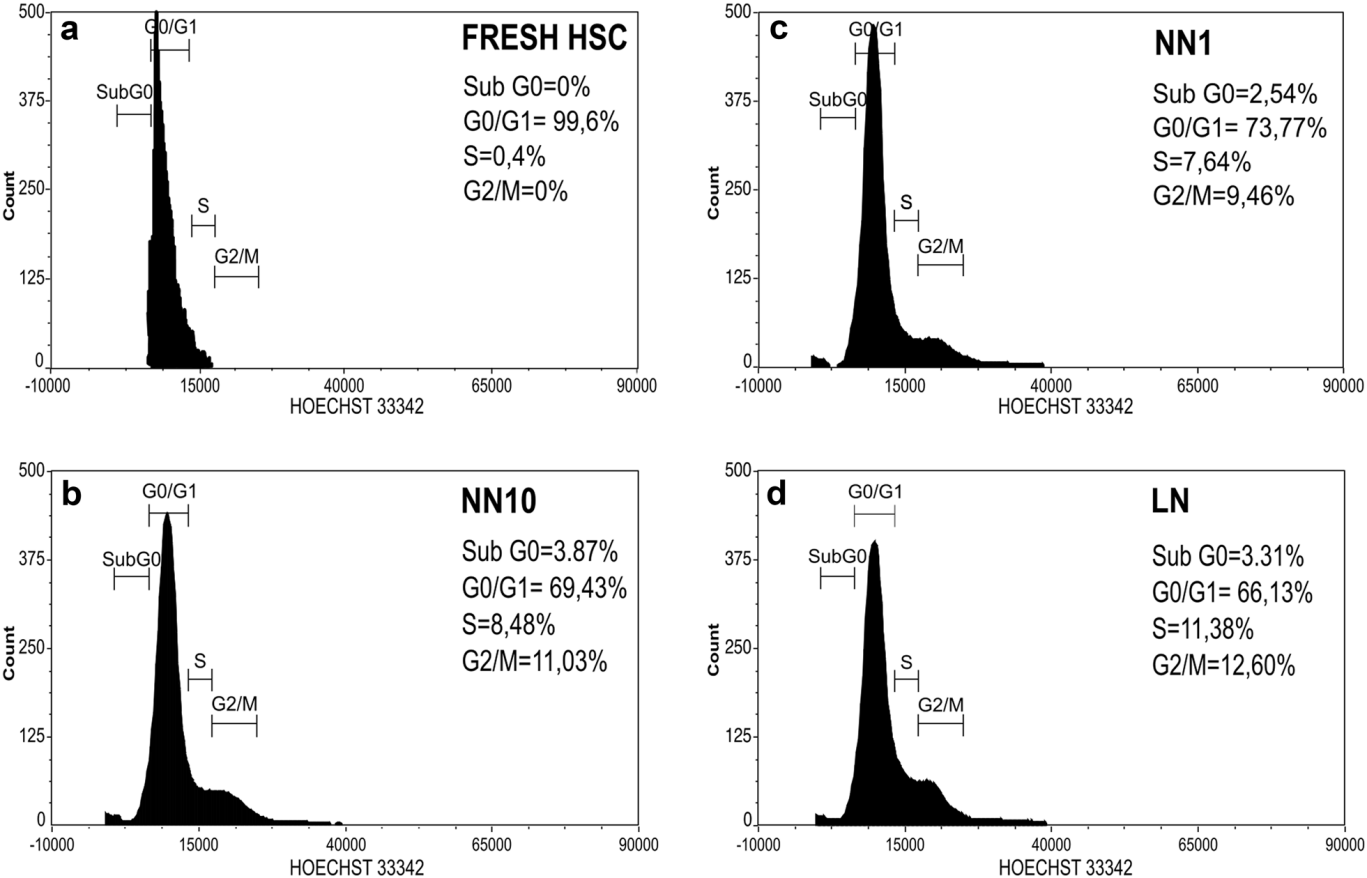
The LN induces increases in the S + G2/M phases of the cell cycle in HSC. Freshly isolated HSC (**a**) or HSC from the different niches (**b** NN10, **c** NN1, **d** LN) were stained with Hoechst 33342 dye for cell cycle evaluation by flow cytometry (FACSAriaTM II, BD Biosciences). Areas under the histograms were used to determine the percentage of cells in sub-G0, G0/G1, S and G2/M phases of the cell cycle. FlowJo and FCS Express Flow Cytometry Data Analysis Softwares v5.0 (BD Biosciences) were used for data analysis. A representative experiment is shown

**Fig. 2:**
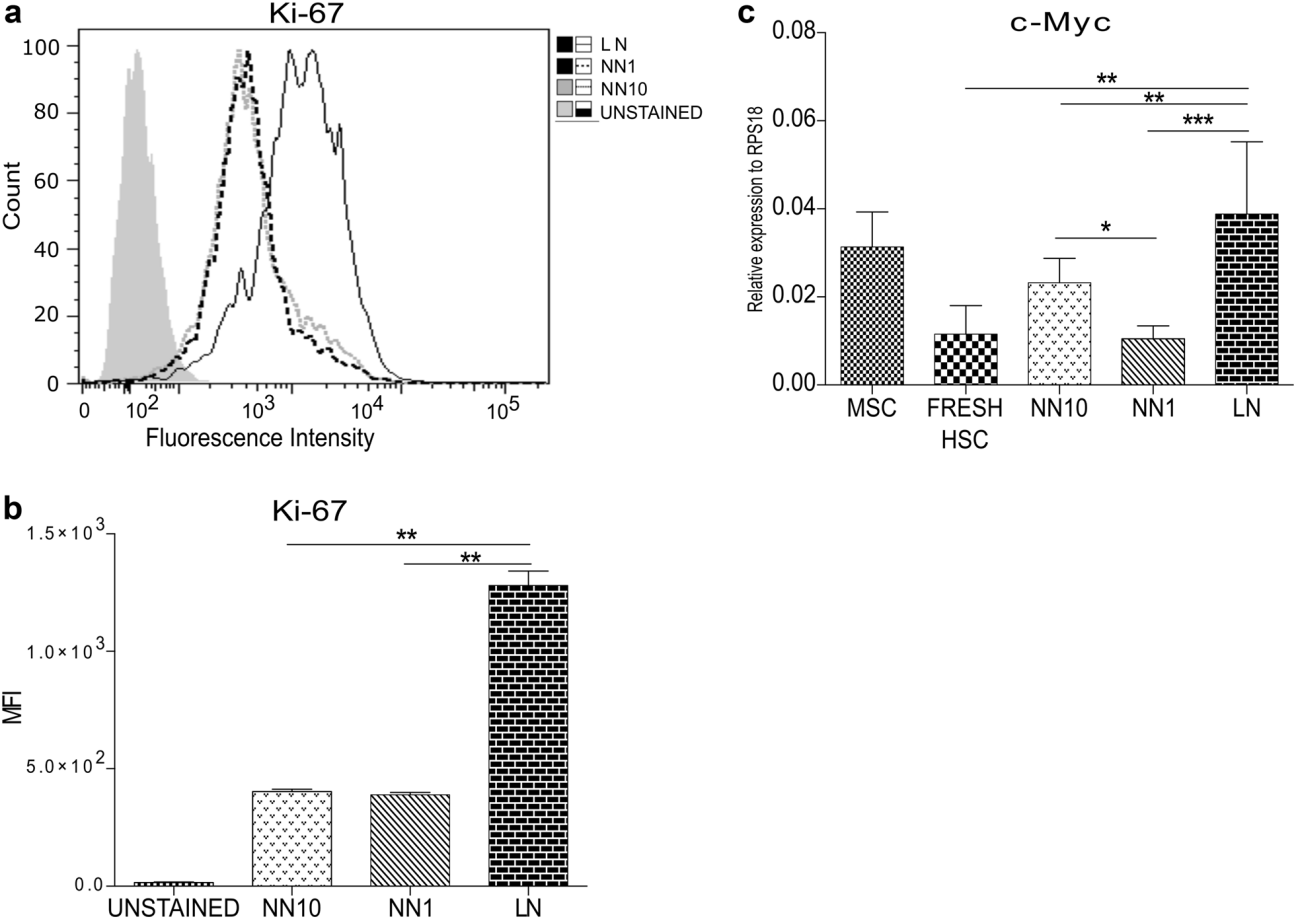
The LN increases proliferation of HSC. HSC proliferation, from the different niches as indicated, was evaluated by labelling cells with anti-Ki67 monoclonal antibody conjugated to FITC by flow cytometry (FACSAriaTM II, BD Bioscience). **a** Representative histogram showing fluorescence intensity of Ki-67. **b** Mean fluorescence intensity (MFI) quantification of HSC in NN10, NN1 and LN stained with anti-Ki-67 antibody. **c** qRT-PCR for the evaluation of c-Myc mRNA expression, as described in “Methods”. FlowJo was used for data analysis. Results shown are from two independent experiments done in duplicates. Error bars represent SEM *p < 0.05;**p < 0.01 (Student’s t test)

We first analyzed Hoechst 33342 staining in freshly isolated HSC and HSC obtained from the different niches (NN10, NN1 and LN). In freshly isolated cells, almost the entire population (99.6%) was in the G0/G1 phase (Fig. 1a) while in the NN10 and N1 this population was near 70% (Fig. 1b, c). As expected, lowering FBS concentration reduced cells in the S- and G2/M phases. Interestingly, we observed a slight increase in the S/G2/M population in HSC obtained from the LN when compared to the NNs (24 vs 17% in NN1 and vs 19.5% in NN10). Considering that after this short period of time (3 days) it is not possible to see big differences in proliferation due to the population doubling (PD) time of HSC in co-culture with MSC, the proliferation-associated nuclear antigen Ki-67 was therefore evaluated. A clear increase of Ki67 in HSC from the LN was observed, while no differences were seen between NN10 and NN1 (Fig. 2a, b). Double labeling with anti-Ki67 and Hoechst 33342 showed clearly that the G0/G1 subpopulation was reduced in the LN compared to NNs (23% vs about 60%, respectively) (Additional file 3: Figure S2). Additionally, the G0 cell subpopulation was reduced in the LN as evaluated by the Ki67 low-expressing cells in the GO/G1 gate. Furthermore, c-Myc expression, a proliferation- and progenitor differentiation-associated transcription factor [32] was increased in the LN (Fig. 2c). Thus, HSC in the LN enter the cell cycle and proliferate, suggesting that the control over cell proliferation, normally exerted by the MSC, is lost in this condition.

### GATA2 y p53 expression in HSC in the LN

**Fig. 3:**
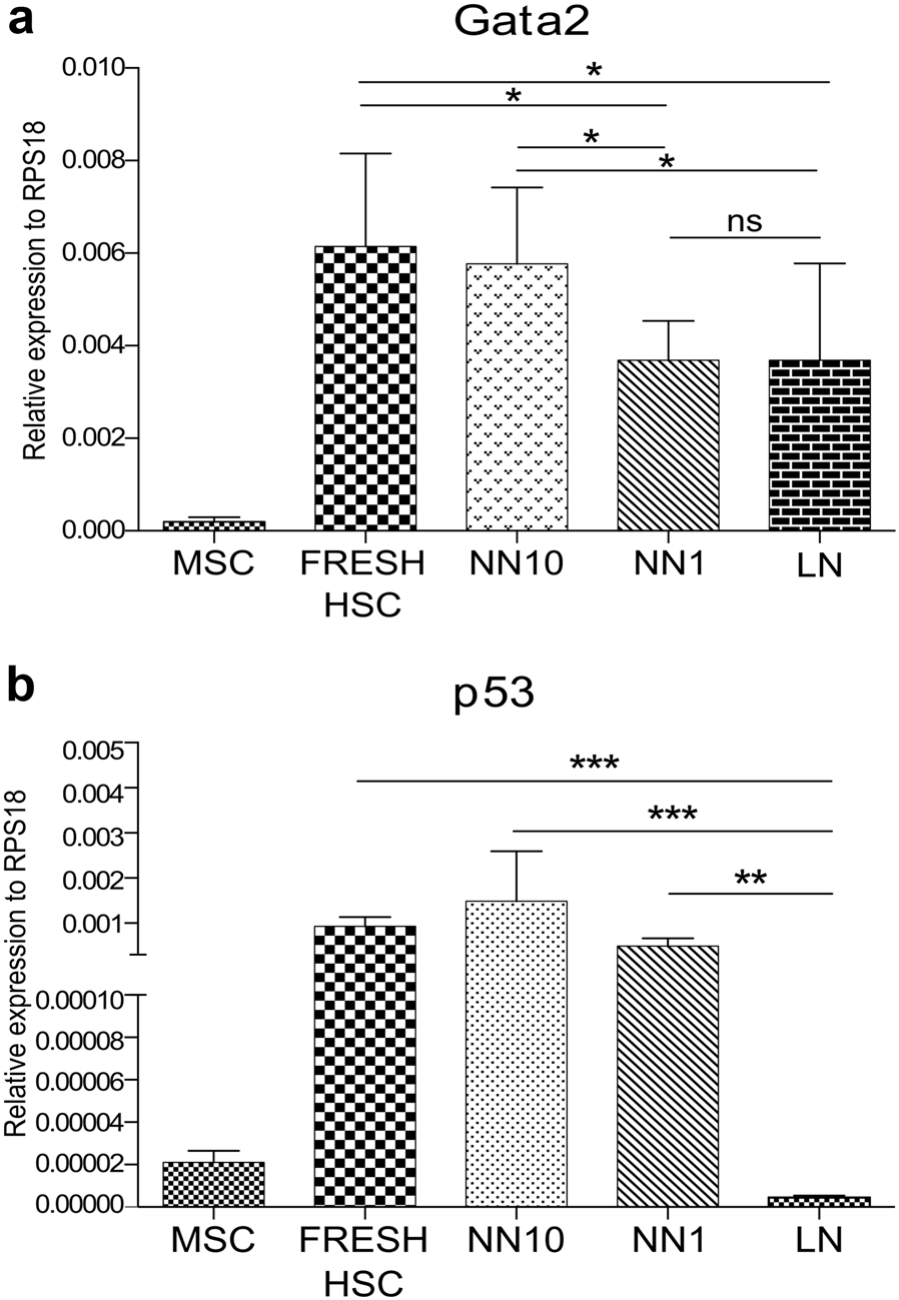
Gata2 and p53 are down regulated in the LN. GATA2 and p53 gene expression was analysed by qRT-PCR in HSC from the different niches and compared to freshly isolated cells. RPS18 gene expression was used to normalize data and samples were run in triplicates (n = 3). **a** Gata2 gene expression. **b** p53 gene expression. Data are shown as mean ± SEM. p values were calculated by paired Student’s t test, (ns: non-significant, *p < 0.05, **p < 0.01)

GATA2 and p53, having overlapped expression patterns [33], are believed to be central regulators of the hematopoietic homeostasis, in special of the HSC quiescence. GATA2 and p53 were expressed in freshly isolated HSC and were not or very low expressed in MSC (Fig. 3a, b) or REH cells (not shown). Compared to NN10, HSC from the LN showed lower GATA2 expression, while no differences were found between NN1 and LN (Fig. 3a). On the contrary, p53 expression was severely reduced in the LN compared to freshly isolated HSC or HSC obtained form NN10 or NN1 (Fig. 3b).

### Expression of genes involved in self-renewal

**Fig. 4:**
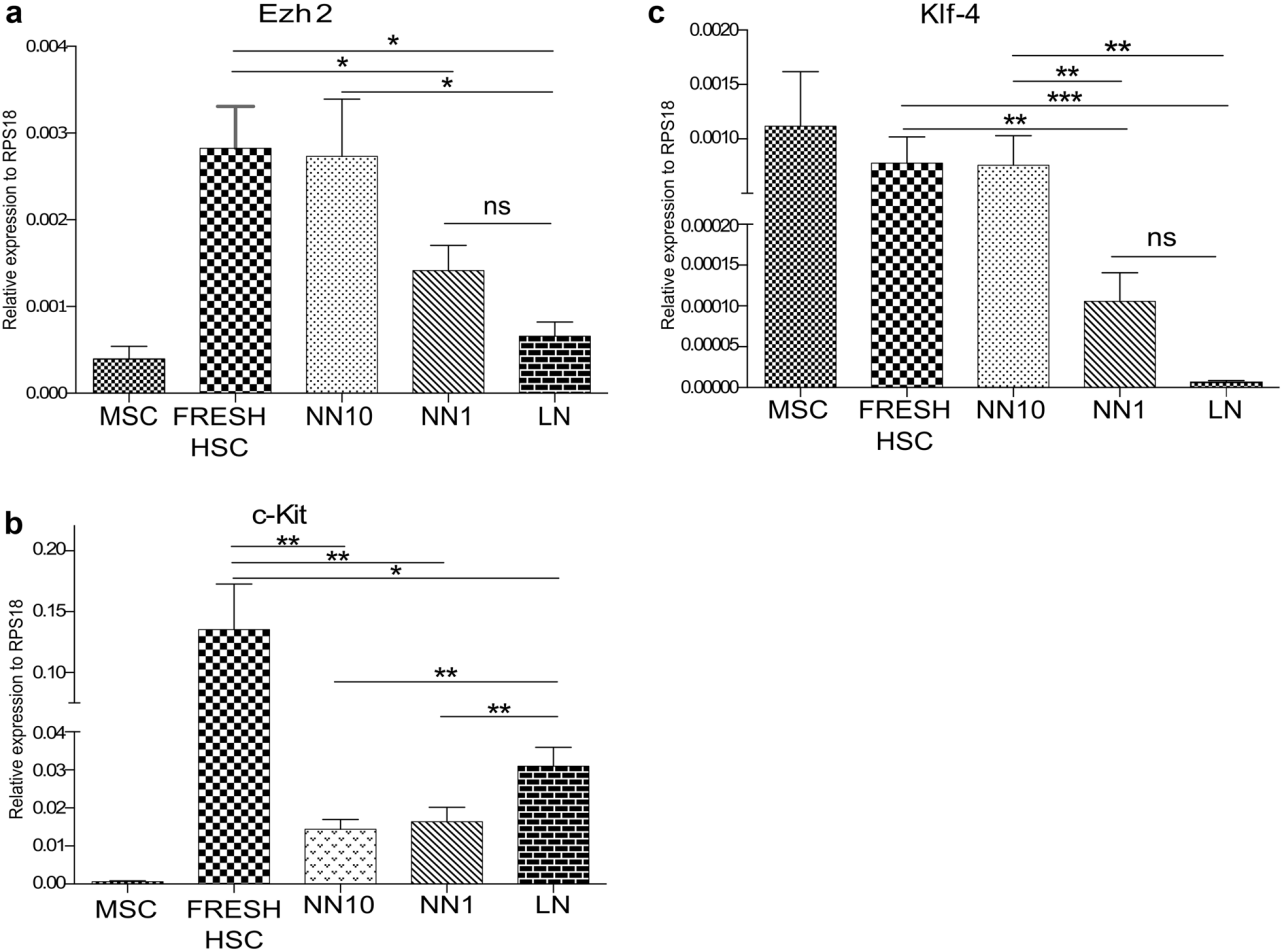
HSC from the LN have abnormal gene expression of molecules involved in self-renewal. RT-qPCR analysis of **a** Ezh2, **b** c-Kit, **c** Klf4 gene expression in HSC co-cultured with MSC and REH-CM. Results are normalized to the housekeeping gene RPS18. Graphs show SEM; n = 3, ns: non-significant, *p < 0.05, **p < 0.01, ***p < 0.001 were calculated by using Student’s t test in GraphPad Prism5

We have also performed a gene expression analysis of other 15 genes supposed to be involved in HSC self-renewal, including Runx1, FoxO3a, SMAD4, Angpt1, Tie2, p16, Notch1, Bmi-1, HoxB4, OCT4 and Nanog [5, 6, 34–38]. None of these 12 genes showed differences in expression between the NN and the LN. Only three genes were differentially expressed in the NN and the LN. Ezh2, a histone–lysine *N*-methyltransferase enzyme, and Klf4, a reprogramming transcription factor, showed both a lower expression in the LN compared to NN10 (Fig. 4a, b). No differences in Ezh2 and Klf4 expression between NN10 and freshly isolated HSC were observed. On the contrary, cKit, the cell receptor for the SCF, had an increased expression (X2) in the LN compared to both NNs (Fig. 4c), but compared to freshly isolated HSC its expression was only one-third. Interestingly, these three genes were highly expressed in fresh HSC, suggesting that a leukemic microenvironment affect their expression importantly.

### The LN induces senescence in MSC and lower migration in HSC

**Fig. 5:**
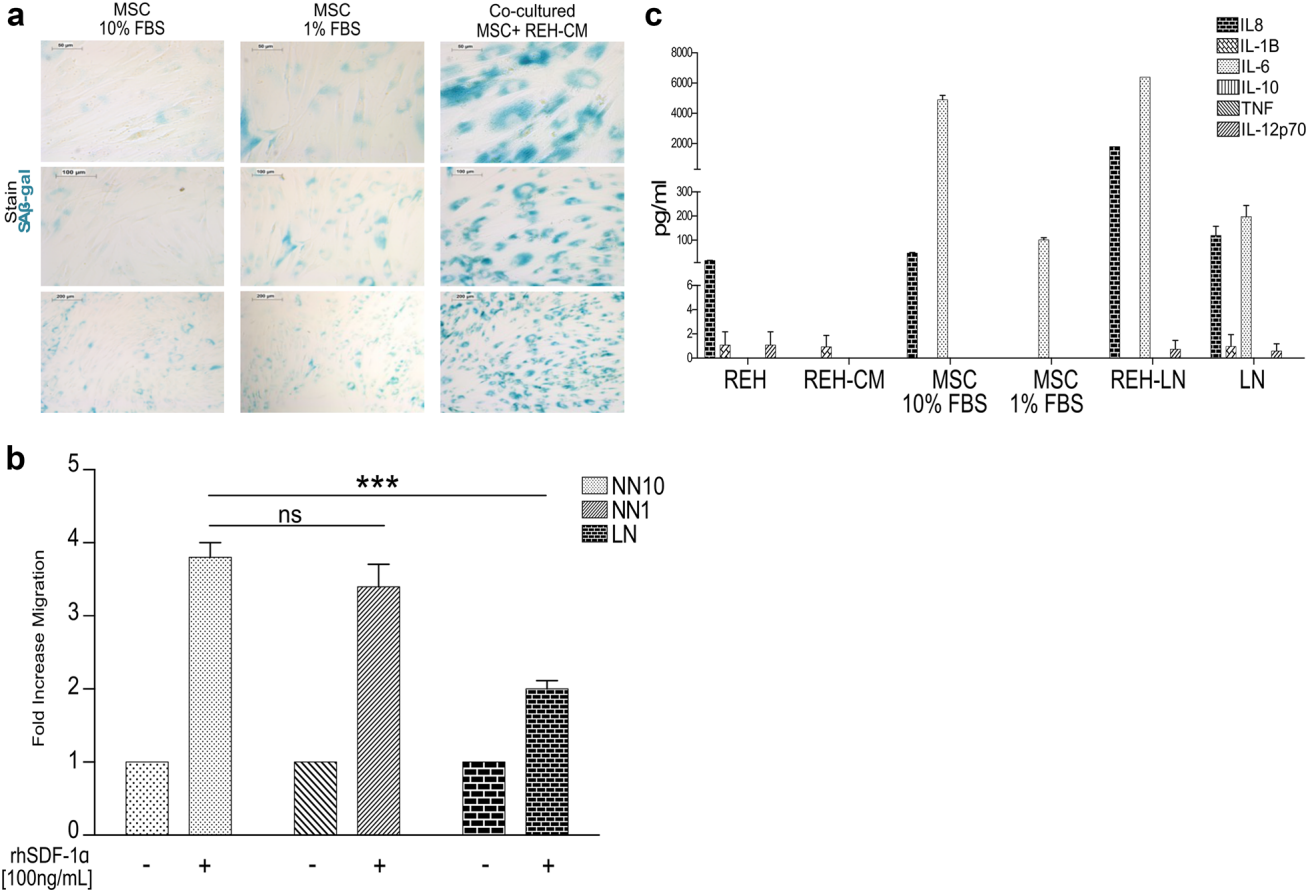
REH-CM increases SA-βGAL activity in the BM-MSC. **a** Representative SA-β-gal staining of BM MSC isolated from the NN10, NN1 and LN. **b** The chemotactic effect of SDF-1 (100 ng/ml) on CD34+ HSC migration in the different niches was studied using a modified Boyden chamber assay. Results are expressed as the fold increase migration from two independent experiments done in duplicates. (ns: non-significant, *p < 0.05, **p < 0.01). **c** Analysis of a multiplexed cytometric bead assay (CBA) of the LN. Cytokines (IL-8, IL-1B, IL-6, IL-10, TNFα and IL-12p70) concentration in the samples was calculated from the data obtained by the FACS array

In the course of the different experiments we observed that after incubation with the REH-CM, MSC stopped dividing and looked flattened with the appearance of small vacuoles. We thus proceeded to evaluate the senescent-associated β-galactosidase (SA-βGal) activity, which was positive after incubation with the leukemic-CM. Control MSC in culture medium were totally normal. This senescent-associated phenotype would explain in part, the loss of cell support of MSC to HSC (Fig. 5a). Since cell cycle entry affects HSC migration capacity [39] we have also study the effect of the LN in HSC migration using a transwell system. Consistently, HSC from the LN migrated less toward SDF-1 (Fig. 5b), showing another functional alteration of HSC in the LN that could be relevant in vivo.

### Partial REH-CM characterization

Since senescence and inflammation are two related processes relevant to tumor growth and progression [40], we studied the inflammatory cytokines that may be present in the LN and responsible for the effects observed in HSC in the LN. Using a CBA assay we were able to show that IL-6 and IL-8 were the most abundant pro-inflammatory cytokines present in the LN (Fig. 5c). These cytokines were not present in the REH-CM itself but were therefore produced by the MSC in the presence of REH-CM. Both cytokines were also induced in MSC by 10% SFB but only IL-6 was induced by 1% SFB. Interestingly, both IL-6 and IL-8 were also detected abundantly when the LN was set with REH cells (REH-LN in Fig. 5c), suggesting that our LN set by incubation of MSC with REH-CM simulates appropriately the leukemic microenvironment.

## Discussion

Here we have shown that MSC control over HSC proliferation is lost after incubation in a leukemic microenvironment. In a short period of incubation in the LN, HSC entered the S/G2/M phase of the cell cycle and increased Ki-67 and c-Myc expression, two well-known markers of cell proliferation. This suggests that HSC in the LN would be in a state prone to further increase proliferation. This is similar to what we have previously reported [31], showing that HSC, in co-culture with MSC, have a lower rate of proliferation than HSC cultured with MSC in the presence of early cytokines (TPO, Flt3L and SCF), strengthening the important role of soluble factors in HSC function. It has been reported that proliferating HSC differentiate into early progenitors [32] and that c-Myc expression is required for cell cycle progression and expansion, contrary to HSC that divide independently of c-Myc. These results are consistent with ours, showing increased c-Myc expression in HSC in the LN.

Early work has suggested a potential link between GATA-2 and p53 pathways [33] and more recently it has been shown that GATA2 and p53 expression are essential regulators of HSC quiescence [41, 42]. Both genes were expressed in freshly isolated HSC and in the NN, but were down regulated in the LN, especially p53. This reduction in GATA2 and p53 would induce the loss of HSC quiescence, in agreement with the increased HSC proliferation observed above. Recently, it was shown that targeting p53 and GATA2 in leukemic stem cells led to synergistic leukemia cell killing without affecting normal HSC [43]. This could probably be explained by lower expression of p53 and GATA2 in HSC in the leukemic microenvironment as found here in our in vitro LN model.

Also, it has been reported that fast-dividing cells have limited self-renewal and reconstitution capabilities [44, 45]. We have also recently shown, that HSC in a leukemic microenvironment set with REH cells have decreased multipotency as evaluated by CFU assays (Vernot JP et al. unpublished). To explore this issue in our LN, we studied genes involved in self-renewal. Unexpectedly, the majority of them were similarly expressed in the NNs and LN with only three genes differentially expressed in these in vitro culture systems. In particular, Klf4 was highly down regulated in the LN compared to HSC from NN or freshly isolated HSC. Also, it has been shown that Klf4 inhibits HSC proliferation by increasing the cell cycle inhibitors p21 and p27 and causing cell cycle arrest in vitro and in vivo [46]. Therefore, its down-regulation is also compatible with HSC increased proliferation. Ezh2 was also down regulated in HSC obtained from the LN. It has been shown that Ezh2 is required for proper B and T cell development [47] suggesting that lower Ezh2 expression would affect differentiation of both lymphocyte subpopulations, similar to the observed effect in leukemia patients. Interestingly, Ezh2 inactivation has been also observed in leukemia [48] and Klf4 may function as a tumor suppressor gene in leukemia [49]. The third gene that we observed differentially expressed in these in vitro systems was c-Kit, the SCF receptor. Though, c-Kit was upregulated in HSC from the LN, but its expression was lower than in fresh HSC. SCF/c-Kit signaling is not only necessary for HSC viability [50], but also for HSC repopulation and self-renewal as evaluated in a non-competitive transplantation assay [18] and for functional positioning HSC in the niche [51]. On the other hand, we have previously shown that lower c-Kit expression reduced HSC multipotency [31]. HSC migration was also affected in HSC obtained from the LN and this could be the consequence of altered adhesion to MSC [13]. Collectively, our results showed that HSC in our in vitro LN lost quiescence, increased the proliferation rate, had reduced self-renewing potential and presented migration defects.

Since in our study we have set the LN without leukemic cells, it is evident that soluble factors are responsible for the observed effects. Our results showed that REH-secreted cytokines are indirectly responsible for the HSC malfunction. In fact, it seems that a senescence process is induced during the setting of the LN, and this is considerably affecting MSC soluble factor secretion. We have performed a preliminary characterization of the soluble factors that could be responsible of the observed effect in the LN. Interestingly, IL-6 and IL-8 were upregulated in the LN but both cytokine were not present in the REH-CM itself, suggesting that their secretion by MSC is induced only by soluble factors secreted by the REH cells. Noteworthy, IL-6 and IL-8 levels increased in serum of leukemic patients and correlated with adverse clinical effects and short survival [52, 53]. Consistently with this, IL-6 and IL-8 were also the major secreted cytokines found in a LN set directly with REH cells. The specific role of these two cytokines alone or together in MSC deserves further experiments. Our increased understanding of the HSC alterations in the LN will foster studies owing to the development of novel therapeutic agents.

## Conclusions

This study validates an in vitro leukemic niche established without leukemic cells to study HSC function, simulating a BM niche affected by the leukemic cells. We showed that HSC increased cell proliferation and presented changes in gene expression that are compatible with the loss of self-renewal potential. This is consistent with reports in patients with ALL, showing reduced hematopoiesis and increased numbers of progenitor and differentiated cells. We showed also that the leukemic microenvironment affected MSC and the soluble factors they secrete, similar to what has been described in patients with leukemia. This in vitro system, set without leukemic cells, may facilitate HSC evaluation and would be a valuable tool for the development of novel therapeutic agents.

## Additional files

**Additional file 1: Table S1.** List of primers used for RT-qPCR.

**Additional file 2: Figure S1.** Isolation and characterization of mesenchymal stem cells and hematopoietic stem cells. A. Flow cytometry analysis showing the expression of MSC surface markers (CD105, CD90, CD73, CD44) in the absence of CD45 and CD34 gene expression (not shown). The percentage of positivity for each marker is indicated. B. In vitro adipo-, chondro- and osteogenic differentiation of BM MSC. C. CD34+ cells were isolated from human UCB by high-gradient magnetic cell sorting. Evaluation of cell purity by flow cytometry of the CD34+ HSC (>90% in all experiments). Results shown are representative of one BM-MSC and one UCB samples.

**Additional file 3: Figure S2.** Double labeling of HSC with Hoechst staining and anti-Ki67 antibody after the co-culture in the NN10, NN1 or LN. Dual-parameter dot plot showing HSC staining with Hoechst and anti-Ki67 antibody, excluding dead cells. Cells in the G0 phase of the cell cycle (low Ki67 expression) appear at the bottom of the G0/G1 gate. Cells in the S/G2 + M phases were increased in the LN. A representative experiment is shown.

## Abbreviations

HSC: hematopoietic stem cells
BM-MSC: bone marrow-mesenchymal stem cells
REH: acute lymphocytic leukemia pre-B (REH) cell line
UCB: umbilical cord blood
VCAM: vascular cell adhesion molecule 1
SDF-1: stromal cell-derived factor 1
ALL: acute lymphoblastic leukaemia
MNC: mononuclear cells
