# Supplementary material for: Loss of quiescence and self-renewal capacity of hematopoietic stem cell in an in vitro leukemic niche
Source: Exp Hematol Oncol. 2017 Jan 10;6:2. doi: 10.1186/s40164-016-0062-1 (PMC5223333; doi:10.1186/s40164-016-0062-1)
Supplement: Supplementary file 1 — Additional file 1: Table S1. List of primers used for RT-qPCR. [file 40164_2016_62_MOESM1_ESM.docx]

| **Supplementary Table 1** List of primers used for RT-qPCR. | | |
| --- | --- | --- |
| Target gene | Oligonucleotide sequences (5’-3’) | Annealing Temperature (ºC) |
| Gata-2 | Forward, 5'-CGTTCCTGTTCAGAAGGC-3';  Reverse, 5'-GTTCTGCCCATTCATCTTGT-3'; | 60 |
| Runx.1 | Forward, 5′- CTGTGTAGGGGAGCCACATT -3′;  Reverse, 5′- CTTGTCTCCACTGAGGCACA -3′; | 60 |
| p53 | Forward, 5′-CTGCCCTCAACAAGATGTTTTG-3′;  Reverse, 5′-CTATCTGAGCAGCGCTCATGG-3′; | 60 |
| Foxo3a | Forward, 5’-TCTACGAGTGGATGGTGCGTT-3’;  Reverse, 5’-CGACTATGCAGTGACAGGTTGTG-3’; | 60 |
| Smad4 | Forward, 5’-ACAAGTCAGCCTGCCAGTATACT-3’;  Reverse, 5’- GGTGGTAGTGCTGTTATGATGGTAAG-3’; | 60 |
| Angpt1 | Forward, 5’-CTCCCTTCCAGCAATAAGTGT-3’;  Reverse, 5’-AGCCCGACAGTCAGTGGAGT-3’; | 60 |
| Tie2 | Forward, 5’-CTGTGAAGGGCGAGTTCGA-3’;  Reverse, 5’-TGGTAGGAAGGAAGCTTGTTGAC-3’; | 60 |
| p16 | Forward, 5’-GAAGGTCCCTCAGACATCCCC-3’;  Reverse, 5’- CCCTGTAGGACCTTCGGTGAC-3’; | 60 |
| EZH2 | Forward, 5’-TTGTTGGCGGAAGCGTGTAAAATC-3’; Reverse, 5’-TCCCTAGTCCCGCGCAATGAGC-3’; | 60 |
| CD34 | Forward, 5’-AAACTACAACACCTAGTACCCTTGGAA-3’;  Reverse, 5’-GAATTTGACTGTCGTTTCTGTGATG-3’; | 60 |
| c-Kit | Forward, 5’-ATTTTCTCTGCGTTCTGCTCCTAC-3’;  Reverse, 5’GCCCACGCGGACTATTAAGTCTGAT-3’; | 60 |
| CXCR4 | Forward, 5’-GCATGACGGACAAGTACAGGCT-3’;  Reverse, 5’-AAAGTACCAGTTTGCCACGGC-3’; | 60 |
| Notch1 | Forward, AATGTGGATGCCGCAGTTGT;  Reverse, GATGTCCCGGTTGGCAAAG; | 60 |
| Bmi-1 | Forward, 5'-TAAGCATTGGGCCATAGT-3';  Reverse,   3'-ATTCTTTCCGTTGGTTGA-5'; | 60 |
| Hoxb4 | Forward, TACCCCTGGATGCGCAAA;  Reverse, CAGGTAGCGGTTGTAGTGAAATTC; | 60 |
| c-Myc | Forward, 5'-GAGCTGTTTGAAGGCTGGATTT-3';  Reverse, 3'-TCCTGTGGTGAAGTTCACGTT-5'; | 60 |
| Oct-4 | Forward, AGTTTGTGCCAGGGTTTTTG;  Reverse, CTTCACCTTCCCTCCAACC; | 60 |
| Klf4 | Forward, TATGACCCACACTGCCAGAA;  Reverse, TGGGAACTTGACCATGATTG; | 60 |
| Nanog | Forward, CCTGTGATTTGTGGGCCT;  Reverse, GACAGTCTCCGTGTGAGGCAT; | 60 |
| RPS18 | Forward, GATGGGCGGCGGAAAATA;  Reverse, GTACTGGCGTGGATTCTGCATA; | 60 |
