# Supplementary figures and images for: Loss of quiescence and self-renewal capacity of hematopoietic stem cell in an in vitro leukemic niche
Source: Exp Hematol Oncol. 2017 Jan 10;6:2. doi: 10.1186/s40164-016-0062-1 (PMC5223333; doi:10.1186/s40164-016-0062-1)

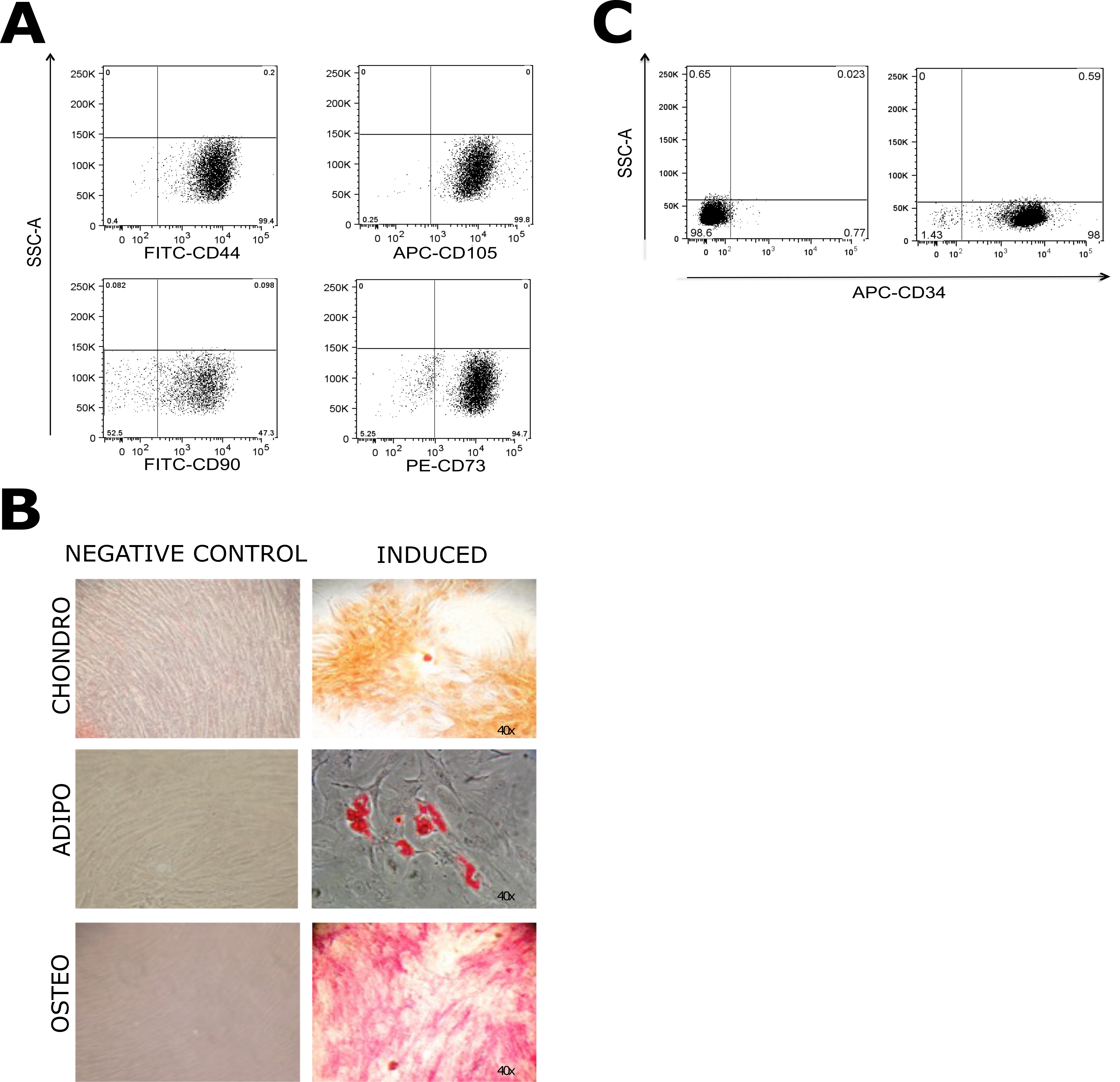

Supplement: Supplementary file 2 — Additional file 2: Figure S1. Isolation and characterization of mesenchymal stem cells and hematopoietic stem cells. A. Flow cytometry analysis showing the expression of MSC surface markers (CD105, CD90, CD73, CD44) in the absence of CD45 and CD34 gene expression (not shown). The percentage of positivity for each marker is indicated. B. In vitro adipo-, chondro- and osteogenic differentiation of BM MSC. C. CD34+ cells were isolated from human UCB by high-gradient magnetic cell sorting. Evaluation of cell purity by flow cytometry of the CD34+ HSC (>90% in all experiments). Results shown are representative of one BM-MSC and one UCB samples. [file 40164_2016_62_MOESM2_ESM.tif]

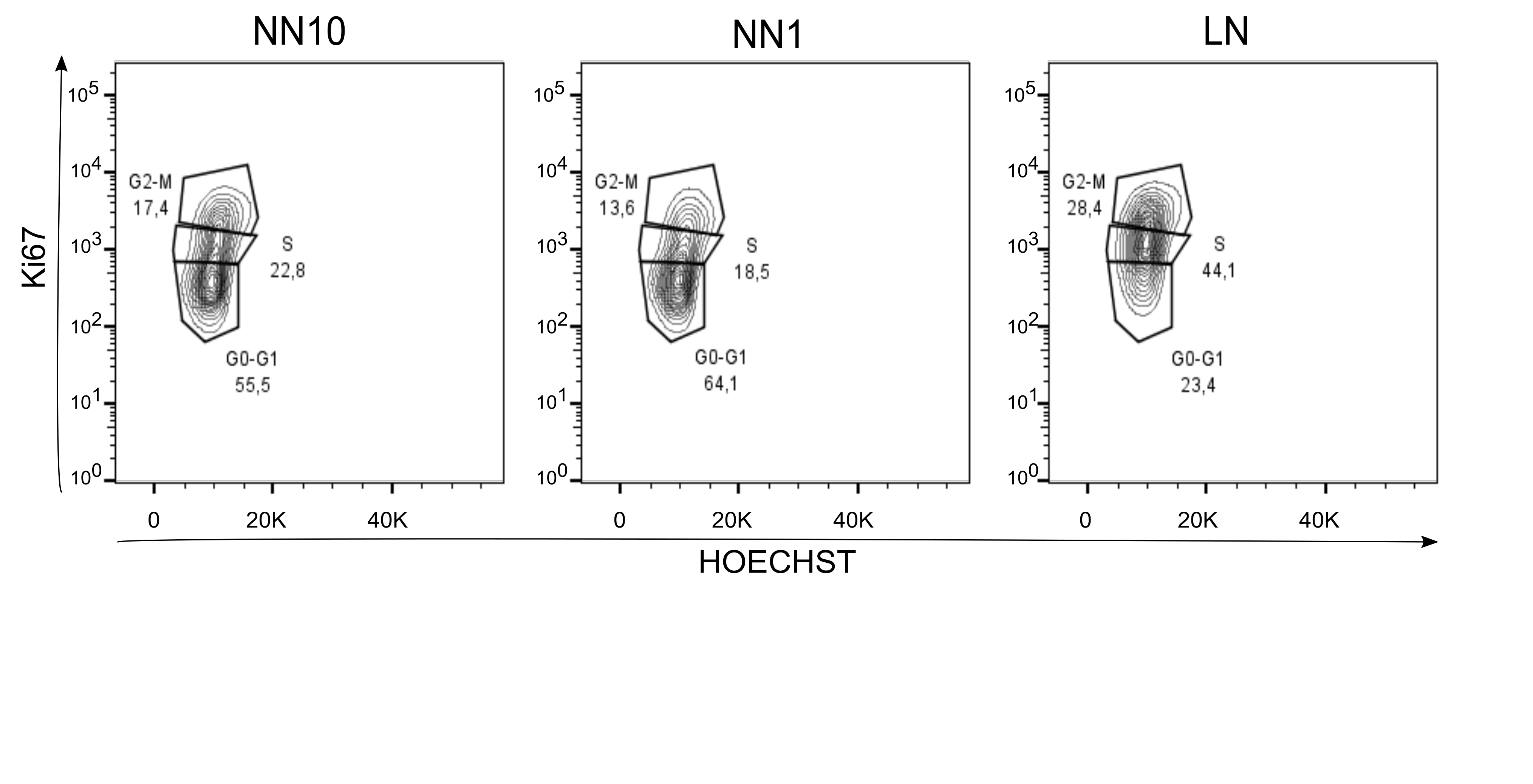

Supplement: Supplementary file 3 — Additional file 3: Figure S2. Double labeling of HSC with Hoechst staining and anti-Ki67 antibody after the co-culture in the NN10, NN1 or LN. Dual-parameter dot plot showing HSC staining with Hoechst and anti-Ki67 antibody, excluding dead cells. Cells in the G0 phase of the cell cycle (low Ki67 expression) appear at the bottom of the G0/G1 gate. Cells in the S/G2 + M phases were increased in the LN. A representative experiment is shown. [file 40164_2016_62_MOESM3_ESM.tif]
